# Chitosan Nanoparticles for Enhanced Immune Response and Delivery of Multi-Epitope *Helicobacter pylori* Vaccines in a BALB/c Mouse Model

**DOI:** 10.3390/pharmaceutics17010132

**Published:** 2025-01-18

**Authors:** Rita Amaral, Tomás Concha, Jorge Vítor, António J. Almeida, Cecília Calado, Lídia M. Gonçalves

**Affiliations:** 1 Research Institute for Medicines (iMed.ULisboa), Faculty of Pharmacy, Universidade de Lisboa, 1649-003 Lisbon, Portugal; ritarlamaral@gmail.com (R.A.); tomas.p.almeida@ff.ulisboa.pt (T.C.); jvitor@ff.ulisboa.pt (J.V.); aalmeida@ff.ulisboa.pt (A.J.A.); 2 ISEL—Instituto Superior de Engenharia de Lisboa, Instituto Politécnico de Lisboa, 1959-007 Lisbon, Portugal; ccalado@deq.isel.pt; 3 iBB—Institute for Bioengineering and Biosciences, i4HB—Associate Laboratory, Institute for Health and Bioeconomy, Instituto Superior Técnico, Universidade de Lisboa, 1049-001 Lisbon, Portugal

**Keywords:** *Helicobacter pylori*, vaccine, DNA vaccine, immunization, recombinant antigens

## Abstract

**Background/Objectives**: *Helicobacter pylori* is the leading cause of chronic gastritis, peptic ulcer, gastric adenocarcinoma, and mucosal-associated lymphoma. Due to the emerging problems with antibiotic treatment against *H. pylori* in clinical practice, *H. pylori* vaccination has gained more interest. Oral immunization is considered a promising approach for preventing initial colonization of this bacterium in the gastrointestinal tract, establishing a first line of defense at gastric mucosal surfaces. Chitosan nanoparticles can be exploited effectively for oral vaccine delivery due to their stability, simplicity of target accessibility, and beneficial mucoadhesive and immunogenic properties. **Methods**: In this study, new multi-epitope pDNA- and recombinant protein-based vaccines incorporating multiple *H. pylori* antigens were produced and encapsulated in chitosan nanoparticles for oral and intramuscular administration. The induced immune response was assessed through the levels of antigen-specific IgGs, secreted mucosal SIgA, and cytokines (IL-2, IL-10, and IFN-γ) in immunized BALB/C mice. **Results**: Intramuscular administration of both pDNA and recombinant protein-based vaccines efficiently stimulated the production of specific IgG2a and IgG1, which was supported by cytokines levels. Oral immunizations with either pDNA or recombinant protein vaccines revealed high SIgA levels, suggesting effective gastric mucosal immunization, contrasting with intramuscular immunizations, which did not induce SIgA. **Conclusions**: These findings indicate that both pDNA and recombinant protein vaccines encapsulated into chitosan nanoparticles are promising candidates for eradicating *H. pylori* and mitigating associated gastric diseases in humans.

## 1. Introduction

*Helicobacter pylori* (*H. pylori*) is a spiral-shaped Gram-negative bacterium and the most common microorganism found in the human stomach. *H. pylori* infection is primarily acquired during childhood, and it can persist as a chronic infection in over 50% of cases [1]. *H. pylori* is highly adapted to the stomach’s acidic environment, using urease secretion to neutralize stomach acid by converting urea to ammonia [2]. Urease, along with other enzymes such as lipases and proteases, disrupts the protective mucus layer, facilitating infection through various virulence factors [3]. The high rate of mutation and recombination, the horizontal transfer of DNA, and the lack of a mismatch repair system of DNA result in high genetic variability among strains. This genetic variability provides strains with an adaptive advantage, which improves the colonization in the gastric environment of the host [4,5]. Virulence factors, including the neuraminil–lactose hemagglutinin connection (HpaA), the vacuolating cytotoxin (VacA), the cytotoxin-associated antigen (CagA), the neutrophil-activating protein (NAP), the Helicobacter outer membrane protein (HomB), chaperonin [GroEL), and B subunit of urease (UreB) [6], contribute to *H. pylori*’s ability to adhere, invade, and persist in the gastric mucosa, which increases the risk of developing severe clinical manifestations. Remarkably, these virulent factors are expressed in a large number of strains and have been shown to be a safe and promising immunization strategy [7,8,9].

Standard treatment for *H. pylori* infection consists of a combination of two antibiotics (e.g., clarithromycin with either amoxicillin or metronidazole) and a proton pump inhibitor (PPI). However, the rise in antibiotic resistance, coupled with poor patient adherence to therapy, may lead to treatment failure, re-infection, and recurrence [10]. Vaccination is, thus, being explored as a strategy to prevent and effectively eliminate the infection, particularly since, despite the body’s strong immune response against *H. pylori*, it fails to fully eradicate the bacteria. This challenge indicates that *H. pylori* has effective mechanisms for evading host defenses and hints at the need to develop vaccine antigens that differ from natural bacterial components to trigger a robust protective response [11].

Recently, DNA vaccination has gained more interest as a strategy for triggering both humoral and cellular immune responses. DNA vaccines offer advantages such as high specificity, stability at room temperature, and low production costs. Furthermore, engineering DNA vaccines to targeted selected epitopes may further enhance safety and efficacy by eliciting a specific immune response to those epitopes [12]. Several experimental DNA vaccines targeting *H. pylori* have shown promising results in preclinical studies [13,14,15,16]. These vaccines, which include monovalent or multi-epitope vaccines, can be delivered with or without specialized delivery systems. These vaccines have demonstrated the ability to elicit significant immune responses, including high levels of antigen-specific immunoglobulins, enhanced cytokine production and gene expression, potent lymphocyte proliferation, and, in some cases, protection from *H. pylori* colonization.

Chitosan (CS), a nontoxic, biodegradable polymer with mucoadhesive and immunoadjuvant properties, has been widely studied for its potential as a carrier [17,18,19] and a vaccine immunoadjuvant (as reviewed by Kurmar et al. [20]. The use of CS nanoparticles offers several advantages, particularly for oral–mucosal vaccination [21,22]. Chitosan-plasmid DNA nanoparticles showed stability during gastrointestinal passage [23], protecting the antigen against enzymatic degradation and gastric harsh conditions, ensuring that the antigen could reach the target with its structural integrity preserved. This is critical for enabling the development of a robust immune response. The nano-scale size of these particles, typically ranging from 200 to 350 nm, enhances uptake by mucosal epithelial cells and antigen-presenting cells [24], facilitating antigen presentation and subsequent immune activation. Moreover, the cationic nature of CS promotes prolonged retention time of the particles on negatively charged mucosal surfaces [23], leading to transfection and immune stimulation [25].

In this study, a multigenic recombinant construct targeting key *H. pylori* virulence factors/antigens (CagA, UreB, HpaA, VacA, GroEL, Homb) was developed and encapsulated in chitosan nanoparticles to produce DNA- and protein-based vaccines. These vaccines were administered orally and intramuscularly (i.m.) in BALB/c mice, and the immune response was evaluated through measurement of the levels of serum-specific IgG, IgG1, and IgG2a, as well as intestinal mucosa secreted IgA and cytokines (IL-2, IL-10, and IFN-γ) from activated splenocytes.

## 2. Materials and Methods

### 2.1. Materials

Low molecular weight (LMW) CS with a degree of deacetylation of 92.2% (50,000–190,000 Da; viscosity of 1% solution in 1% acetic acid: 42 cP) sodium alginate (Alg), sodium deoxycholate (DS), and sodium tripolyphosphate (TPP) were purchased from Sigma Aldrich (Dorset, UK).

### 2.2. Animals

Nine groups of female BALB/c mice (Charles River Laboratories, Saint Germain Nuelles, France) (*n* = 5/group), 6–8 weeks old and provided with food and drink ad libitum, were used in the in vivo studies, all experiments were carried out in accordance with the animal welfare body of the Faculty of Pharmacy, University of Lisbon, approved by the competent national authority (Direção Geral de Alimentação e Veterinária—DGAV) under the project PTDC/BIO/69242/2006, which were performed in strict accordance with the EU Directive (2010/63/EU), Portuguese laws (DL 113/2013, 2880/2015, 260/2016 and 1/2019) and all relevant legislation.

### 2.3. Construction and Expression of Recombinant Protein

The following *H. pylori* virulence factors were considered: CagA, UreB, HpA, VacA, GroEL, and Homb. A sequence of 70 amino acid residues from each protein was selected, as presenting high antigenicity as predicted by Jameson–Wolf, Rothbard–Taylor, and AMPHI methods (Lasergene^®^ ProteanTM software V8, DNASTAR Inc., Madison, WI, USA), and its corresponding gene conservation based on the equivalent sequences from different *H. pylori* strains deposit in the EMBL Nucleotide Sequence Database using the Epitope conservancy analysis tool (http://tools.immuneepitope.org). The gene sequences corresponding to 70 amino acid residues from each target were separated by each other with a corresponding flexible linker ([GGGGS]_2_) and a C-end reporter sequence of DYKDDDDK. The whole construction was synthesized, codon-optimized by Geneart (Regensburg, Germany), and cloned at pQE30 (QIAGEN GmbH, Hilden, Germany) and pVAX1 (Invitrogen, Paisley, UK) plasmids.

#### Cell Transformations and Plasmid Production and Purification

*Escherichia coli* (*E. coli*) XL1-Blue cells (Stratagene, La Jolla, CA, USA) were transformed with two plasmid vectors pQE30 (CUHpVGHO) (QIAGEN GmbH, Hilden, Germany) for protein expression and pVAX1 (CUHpVGHO) (Invitrogen, Paisley, UK) for plasmid production, containing the recombinant gene for the following fragment antigens of *H. pylori* CagA, UreB, HpaA, VacA, GroEL, Homb (CUHpVGHO) using CaCl_2_ and heat shock (42 °C for 90 s) treatment [15]. The positive clones were selected in agar with 100 µg/mL ampicillin for clone 6T and 25 µg/mL kanamycin for 6T-plasmid, and a cell bank was produced and preserved with 20% glycerol at −80 °C for each clone. After culture of the selected 6T-plasmid colony on tryptic soy broth medium (Biokar, Allonne, France) containing 25 µg/mL kanamycin antibiotic at 37 °C under agitation (250 rpm) for 10 h, the plasmid purification was carried out using Mega QIAfilter Plasmid purification kit (QIAGEN GmbH, Hilden, Germany) according to manufacturer recommendations. The quantification of the plasmid was assessed by Quant-iT™ Picogreen^®^ assay kit (Molecular Probes™, Invitrogen, Paisley, UK) according to the manual instructions (PicoGreen^®^ dsDNA Quantification Reagent and Kits), and the fluorescence was measured at 480 nm excitation wavelength and 520 nm emission wavelength in a fluorescence microplate reader (Infinite M200, Tecan, Grödig, Austria).

### 2.4. Protein Production and Purification

The protein production was performed according to a procedure previously optimized for histidine (Hist)-tagged recombinant protein produced by *E. coli* under denatured conditions [26]. Briefly, *E. coli* XL-1 Blue expressing pP-CUHpVGHO protein was expanded using tryptic soy broth culture medium (Biokar, Allonne, France) containing 100 µg/mL of ampicillin at 37 °C. After induction with 1 mM IPTG (isopropyl b-D-1-thiogalactopyranoside), bacteria expressing the pP-CUHpVGHO protein were harvested by centrifugation. The cell pellets were homogenized in lysing buffer (20 mM sodium phosphate, 500 mM NaCl, 0.5% (*v*/*v*) Triton X-100, 8 M Urea, pH 8.0) with a tip sonicator for 3 cycles of 5 min at 20% of potency (Branson Sonifier 250, Connecticut, USA) in an ice bath. The insoluble material was removed by centrifugation at 30,000× *g* for 30 min at 4 °C (Beckman 64R, Indianapolis, IN, USA). The recombinant protein was purified using immobilized metal ion affinity chromatography (IMAC) in Hist Trap FF 1 mL nickel column (GE Healthcare, Amersham, UK) under denaturing conditions. The purified protein from the 1 mL Nickel column was desalted on a Sephadex G-25 medium pre-filled PD-10 column (GE Healthcare) using 100 mM Hepes buffer (pH 7.4). Finally, samples of the purified protein were stored at −80 °C or lyophilized and stored at 5 °C.

### 2.5. Protein Analysis and Quantification

The eluted proteins were analyzed on 12% (*w*/*v*) SDS–PAGE under reduced conditions, immunoblotted using anti-Hist antibody following the NuPAGE Novex Bis-Tris Mini Gels Technical Guide (Invitrogen, Paisley, UK), and further quantified by the BCA Protein Assay kit (Pierce, Thermo Fisher Scientific Inc., Waltham, MA, USA) using BSA as a standard.

Protein samples from SDS-PAGE were transferred onto a polyvinylidene fluoride (PVDF) membrane using a semi-dry transfer system (Hoefer Semiphor Amersham, GE Healthcare Life Science, Amersham, UK) at 0.8 mA/cm2 for 1 h. Nonspecific binding sites were blocked using a blocking buffer (10% dry milk in PBS, pH 7.4, with 0.05% Tween 20) for 1 h under constant agitation. The membrane was incubated for 2 h at room temperature with mouse anti-6XHist tag protein (Sigma Aldrich Co., Madrid, Spain) diluted 1:1000 in the blocking buffer under constant agitation. After washing, the membrane was incubated with a goat anti-mouse IgG conjugated to phosphatase alkaline (Sigma Aldrich Co., Madrid, Spain) and incubated for 1 h at room temperature. Binding was detected using the substrate kit of alkaline phosphatase SIGMA FAST™ BCIP (5-bromo-4-chloro-3′-indolyphosphate p-toluidine salt) and NBT (nitro-blue tetrazolium chloride) (Sigma Aldrich Co., Madrid, Spain) in accordance with the kit instructions.

### 2.6. Synthesis of Chitosan Nanoparticles Using Ionic Gelation Method

For the i.m. immunization, the recombinant protein or the plasmid was incorporated into CS/DS (1:1 *w*/*w*) nanoparticles that were prepared using the ionic gelation method, as described by Cadete et al. [27]. Briefly, chitosan and sodium deoxycholate were separately dissolved in highly purified water to obtain a 1 mg/mL solution. Nanoparticles were formed when one volume of DS solution was added dropwise to one volume of CS solution and magnetically stirred at 300 rpm for 15 min at room temperature. For plasmid and protein nanoencapsulation, plasmid or protein was added to DS solution before the addition to CS solution.

For oral vaccination, the antigens, recombinant protein, or plasmid were encapsulated in CS/TPP/Alg (20:0.25:1 *w*/*w*/*w*) nanoparticles. Those nanoparticles were prepared using the method described by Gonçalves et al. [28]. CS, TPP, and Alg (low viscosity) were separately dissolved in highly purified water to obtain 2.5 mg/mL, 10 mg/mL, and 3 mg/mL solutions, respectively. Alg and TPP were then mixed and added dropwise to the CS solution. For plasmid and protein nanoencapsulation, plasmid or protein was added to Alg/TPP solution before the addition to CS solution.

### 2.7. Encapsulation Efficiency

For nanoparticles encapsulating recombinant protein, the encapsulation efficiency percentage (%EE) was indirectly determined by quantifying the concentration of recombinant protein present in the sample supernatants. Briefly, each nanoparticle sample was centrifuged (MiniSpin^®^, Eppendorf, Enfield, CT, USA) at 13,400× *g* for 10 min at room temperature to separate the nanoparticles from the unencapsulated recombinant protein in the supernatant. The protein concentration in the supernatant was measured using the Pierce™ BCA Protein Assay kit (Thermo Fisher Scientific, Rockford, IL, USA) following the manufacturer’s instructions. The free recombinant protein concentration was calculated by linear regression, and the %EE was calculated using Equation (1).

(1) $$\%EE=\frac{{\left[rProtein\right]}_{total}-{\left[rProtein\right]}_{supernatant}}{{\left[rProtein\right]}_{total}}\times 100$$

Similarly, for the nanoparticles with plasmid encapsulated, the %EE was indirectly calculated, this time measuring the concentration of DNA present in the supernatant. Each sample was centrifuged at 13,400× *g* for 10 min at room temperature, and the DNA was quantified as previously explained in Section 2.3. %EE was calculated using Equation (2).

(2) $$\%EE=\frac{{\left[pDNA\right]}_{total}-{\left[pDNA\right]}_{supernatant}}{{\left[pDNA\right]}_{total}}\times 100$$

### 2.8. Nanoparticle Size Characterization

Mean particle size was determined by dynamic light scattering (Zetasizer Nano-S, Malvern Instruments, Malvern, UK) at 25 °C, with a scattering angle of 173°. In all cases, samples were measured in triplicate, and results are presented as mean ± standard deviation (SD).

### 2.9. Immunization Studies

Nine groups of female BALB/c mice (*n* = 5/group) of approximately equal ages and weights (6 to 7 weeks of age) were immunized, either by the oral (intragastric gavage) or the i.m. route, on day 1 and boosted on day 22. Each mouse received 50 μg of nanoencapsulated plasmid in nanoparticles or 50 μg of nanoencapsulated protein in nanoparticles. Control groups were administered with equivalent doses of non-encapsulated plasmid, protein, or empty nanoparticles in Hepes buffer. The immunization groups were divided, as shown in Table 1.

### 2.10. Immune Response Evaluation

Blood samples of each mouse were collected from the tail vein after the first immunization every 2 weeks for 4 months. Samples were centrifuged at 18,000× *g* for 20 min at 4 °C, and collected sera were stored at −20 °C until analysis. Serum-specific antibody (IgG, IgG1, and IgG2a) levels were assessed by the enzyme-linked immunosorbent assay (ELISA), as it was described elsewhere [29]. ELISA plates were coated with the recombinant protein (5.0 μg/mL) at 4 °C overnight and blocked with 5% skimmed milk powder (Merck KGaA, Darmstadt, Germany). Plates were washed again, and sera were tested by serial two-fold dilutions. Sera obtained from naive mice were used as a control. Horseradish peroxide conjugated goat anti-mouse IgG (Sigma-Aldrich Co., Ltd., Dorset, UK), IgG1, and IgG2a (Serotec, Kidlington, Oxfordshire, UK) (diluted 1:1000) were applied as secondary antibody. Finally, the substrate OPD (SigmaFAST™ OPD Kit, Sigma Aldrich Co., Madrid, Spain) was used to develop the plates. The color reaction was stopped after 15 min by adding 2.5N H_2_SO_4_ to the wells, and the absorbance was measured at 490 nm. Titers reported are the reciprocal of serum dilutions that gave an optical density (OD) 5% higher than the strongest negative control reading.

To assess intestine IgA levels, mice were sacrificed, and the intestines were homogenized in 0.9% sodium chloride with protease inhibitor 1 mM PMSF and 0.5% Tween^®^ 20. Homogenized tissues were centrifuged, and the supernatants were freeze-dried. Subsequently, samples were reconstituted with 500 μL of sterile water and added directly in triplicates to the plate wells previously adsorbed with the recombinant protein (5.0 μg/mL). Horseradish peroxide conjugate goat anti-mouse IgA (1:2000; Serotec, Kidlington, Oxfordshire, UK) was used to detect this antibody, and OPD substrate was then added, as mentioned above. The mean OD was determined for each treatment group and used directly to compare mucosal response.

### 2.11. Cytokine ELISAs

The levels of IL-2, IL-10, and IFN-γ were measured by antigen-capture ELISA. The spleens were aseptically removed 15 weeks after mice immunization, and a spleen cell suspension was prepared, as previously described by our group [20]. Briefly, splenocytes were adjusted to a concentration of 2 × 10^6^ viable cells per 100 μL in RPMI complete medium. Cell suspensions were cultured in 96 well plates (Greiner Bio-One GmbH, Kremsmünster, Austria) containing 100 μL of RPMI complete medium and other plates containing 5 μg/mL of recombinant protein (pP-CUHpVGHO). Plates were incubated in a 5% (*v*/*v*) CO_2_ incubator for 72 h at 37 °C to induce cytokines expression in vitro. At the end of incubation, the supernatants were collected, and cytokines were quantified by ELISA sandwich using the mouse IL-2, IL-10, and IFN-γ ELISA kits (RayBiotech, Peachtree Corners, GA, USA) following manufacturer instructions.

### 2.12. Statistical Analysis

Results are expressed as mean ± SD. Statistical analysis was conducted using GraphPad Prism software (version 5.0, Boston, MA, USA). Raw data were first checked for normality using the Shapiro–Wilk test. Normally distributed data were analyzed using the Student’s *t*-test for comparisons between two groups and ANOVA for comparisons involving more than two groups. Non-normally distributed data were analyzed using the nonparametric Mann–Whitney test for comparisons between two groups and the Kruskal–Wallis test, followed by Dunn’s test for multiple comparisons for comparisons involving more than two groups. *p*-values < 0.05 were considered statistically significant.

## 3. Results

### 3.1. Plasmid and Multi-Epitope Protein Production

**Figure 1:**
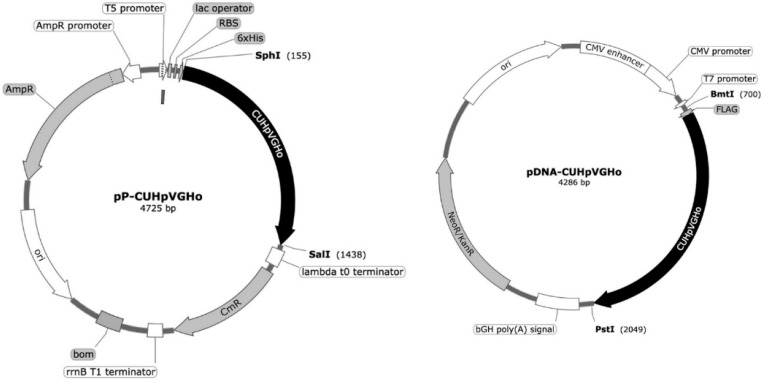
*H. pylori* DNA and protein vaccines. Schematic representation of the *H. pylori* vaccine construction (pP-CUHpVGHO in pQE30 and pDNA-CUHpVGHO in pVAX1).

*E. coli* XL1-Blue cells were transformed with two plasmids, pQE30 (pP-CUHpVGHO) and pVAX1 (pDNA-CUHpVGHO), to produce the recombinant protein and plasmid, respectively, used as vaccine antigens. The genetic sequence cloned into the plasmid vectors, coding for the recombinant protein and pDNA, contains the immunogenic and conserved sequences of the following *H. pylori* antigens: CagA, UreB, HpaA, VacA, GroEL, and Homb (Figure 1).

To determine the suitable stage of cell cultivation for optimal expression of the recombinant protein (pP-CUHpVGHO), *E. coli* cultures were incubated in a shake flask at 37 °C. Sampling was performed each 1 h, and the culture growth was followed by measuring the OD at 610 nm over 24 h of incubation. The culture begins the exponential growth after 5 h of incubation, where the OD 610 nm reaches the value of 0.600 u.a. (units of absorbance). At this stage, the cells initiate a period of exponential cell division and consequently start protein synthesis.

**Figure 2:**
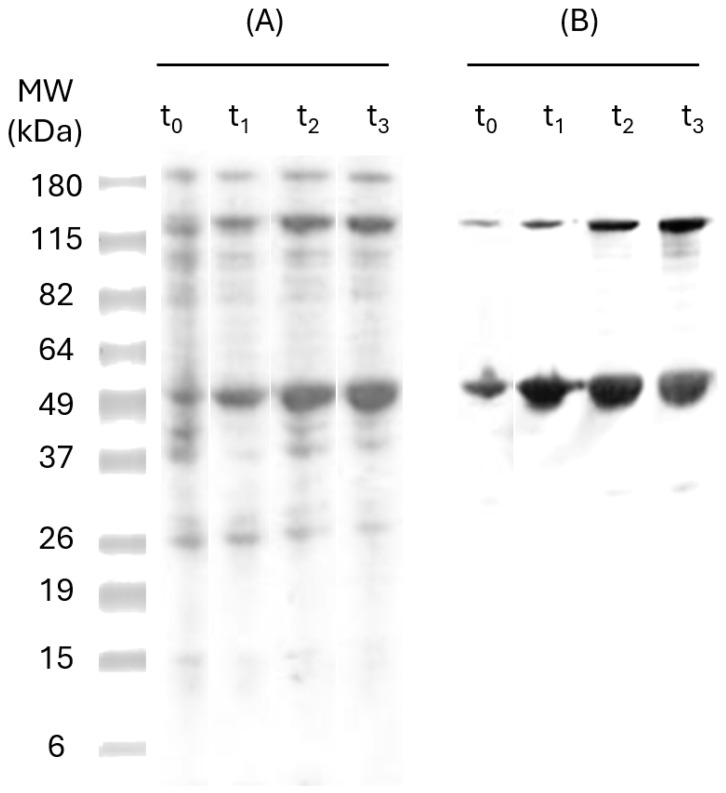
Total protein analysis of *E. coli* XL1 Blue expressing pP-CUHpVGHO recombinant protein by SDS-PAGE (**A**) and Western Blot (**B**) with anti-HisTag antibodies. MW—pre-stained molecular weight markers; t_0_—sample before induction with IPTG; t_1_—sample after 1 h of induction with IPTG; t_2_—sample after 2 h of induction with IPTG; t_3_—sample after 3 h of induction with IPTG.

To determine the ideal incubation time with IPTG, SDS-PAGE and Western Blot analyses were performed for the samples taken over 3 h (time 0, 1, 2, and 3) post-induction. The results (Figure 2) have shown a band of approximately 47 kDa, which is similar in size to the recombinant protein (47 kDa). The six-Hist tag on pP-CUHpVGHO recombinant proteins allows detection of the protein by anti-HistTag antibodies in Western Blot, indicating effective induction and expression [30]. Another band of 115 kDa was observed, corresponding to the oligomeric fraction of the pP-CUHpVGHO recombinant protein. The intensity of both bands has increased over time, and no more expression was noticed after 3 h of induction since there was no significant difference in the amount expressed between t_2_ and t_3_. Therefore, an optimal 3 h incubation time after IPTG induction was established to maximize protein expression.

### 3.2. Protein Purification and Quantification

**Figure 3:**
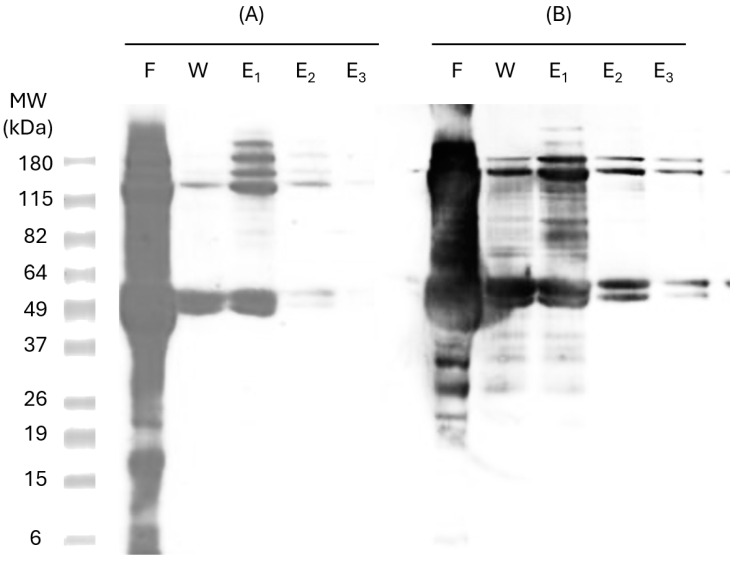
SDS-PAGE 10% polyacrylamide gel (**A**) and Western Blot (**B**) of samples from purification process of recombinant protein. MW—molecular weight standards; F—flow-through sample; W—wash sample; E_1_—first elution sample; E_2_—second elution sample; E_3_—third elution sample.

The expressed proteins were purified through IMAC in a Hist Trap FF 1 mL nickel column (GE Healthcare, Amersham, UK). Six consecutive Hist residues tagged to the recombinant proteins allow specific binding to metal ligands, such as nickel, enabling the protein to be retained on the column. This procedure was performed under denaturing conditions (8M urea). To confirm protein identity in the purified samples, SDS-PAGE and Western Blot analyses were carried out using specific anti-Hist antibodies (Figure 3), confirming the presence of the two bands corresponding to pP-CUHpVGHO recombinant protein (47 kDa and 115 kDa).

Heterologous proteins produced with 6-Hist-tagged recombinant proteins in *E. coli* may experience misfolding and form oligomers, which also may occur from Hist tails. These oligomers are difficult to remove even under denaturing conditions (8 M urea), as observed in the 115 kDa band. Analysis of the nickel column flow-through (Lane F, Figure 3) indicates the presence of other bacterial proteins. To remove non-specifically bound proteins and minimize host cell protein binding, the column was washed with a binding buffer containing a low concentration of imidazole (30 mM). However, imidazole itself may impair the binding of some pP-CUHpVGHO proteins, leading to protein loss during the washing step (Lane W, Figure 3). The recombinant protein was successfully purified with a high purity level with the three elution steps since no other contaminants were detected in the Western Blot (Lanes E_2_ and E_3_, Figure 3). The next step of purification was the buffer exchange using a P10 desalting column.

The purified protein was quantified using the BCA protein assay [31], which presents the advantage of being unaffected by denaturing agents such as urea [32], so it is appropriate for pP-CUHpVGHO recombinant protein quantification in urea (8 M). Protein yield after purification was 58% (Table 2).

### 3.3. Plasmid Purification and Quantification

Large-scale production of pDNA for vaccine formulation requires it to be a highly purified molecule without any traces of RNA, endotoxins, and proteins. Accordingly, anion-exchange chromatography provides a good selectivity for pDNA purification in the presence of RNA, endotoxins, and residual protein [33]. To evaluate plasmid yield after purification, samples were collected during the plasmid DNA purification procedure and analyzed by electrophoresis in 1% agarose gel stained with ethidium bromide. Under certain conditions, plasmid DNA can assume other isoforms, such as the supercoiled form, which tends to migrate faster than circular plasmid DNA [34].

**Figure 4:**
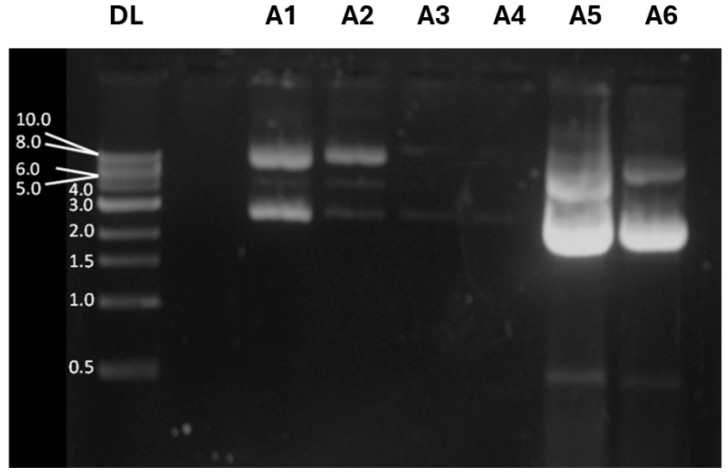
Confirmation of plasmid DNA purification by agarose gel electrophoresis. DL—DNA ladder containing the molecular weight standards. A1—sample of cleared lysate; A2—sample from the flow-through faction; A3—sample from washing; A4—sample from the eluate; A5—sample obtained after precipitation with isopropanol; A6—sample obtained after washing with 70% ethanol.

Figure 4 shows a filtered lysate (A1) containing supercoiled and open circular plasmid DNA, along with other isoforms of supercoiled plasmid DNA, as expected; fraction A2 contains faint bands corresponding to different pDNA isoforms that do not bind to the QIAGEN resin, while fraction A3 reveals the sample after washing the QIAGEN resin with a specific buffer to remove all contaminants. These results indicate no pDNA loss during purification and confirm that the pDNA is free of contaminants. Sample A4 corresponds to the elution fraction of DNA before precipitation with isopropanol, where pDNA is not observed. However, the samples A5 and A6, taken after precipitating the purified DNA with isopropanol, display strong bands at 4.0 kb and at 2.0 kb, corresponding to circular and supercoiled forms of the pDNA-CUHpVGHO, respectively. The intensity measurement of the emitted fluorescence confirmed that a large amount of pDNA was purified with success in both circular and supercoiled forms.

Quant-iT™ Picogreen^®^ dsDNA was used to quantify plasmid DNA concentration [35]. The purified plasmid amount precipitated after centrifugation and redissolved afterward from tube walls (plasmid residual) was also quantified (Table 3).

### 3.4. Nanoparticle Characterization

Two types of CS nanoparticles were formulated for plasmid or protein incorporation: CS/DS nanoparticles for i.m. vaccination and CS//TPP/Alg nanoparticles for oral immunization. Physicochemical properties of nanoparticles, including particle size and encapsulation efficiency, were evaluated, as was described elsewhere [27], and are summarized in Table 4. For CS/DS nanoparticles, additional characterization data are available in the work of Cadete et al. [27], where the same formulation was used. In that study, atomic force microscopy (AFM) confirmed that size values obtained via AFM were consistent with those from dynamic light scattering analysis. The nanoparticles exhibited a spherical morphology with an irregular and rough surface, likely due to pDNA-CS and CS-DS interactions. The polydispersity index of these nanoparticles was reported as 0.275, indicating a moderate size distribution. Although it may be inferred that CS/TPP/Alg nanoparticles could exhibit similar spherical morphology due to a comparable preparation process, this cannot be confirmed since no microscopy images or detailed morphological characterization of the nanoparticles used for oral immunization were available in the present study [27].

### 3.5. Immune Response Elicited by Immunization with Nanoparticles with Recombinant Protein pP-CUHpVGHO or Plasmid pDNA-CUHpVGHO

To measure and evaluate the immune response elicited by recombinant proteins and pDNA, BALB/c mice were immunized twice by oral and i.m. routes with recombinant proteins or pDNA, either nanoencapsulated or in solution. Mice received a priming dose on day 1 and a booster dose on day 22, with no adverse reactions (e.g., lethargy or weight loss) being observed throughout the experiment duration. Specific IgG isotypes (IgG1 and IgG2a), indicative of Th2 and Th1 responses, respectively, were evaluated in sera to assess whether the administered vaccines elicited a balanced immune response. Sera from negative controls (group 9) provided baseline titer values for comparison with treated groups.

**Figure 5:**
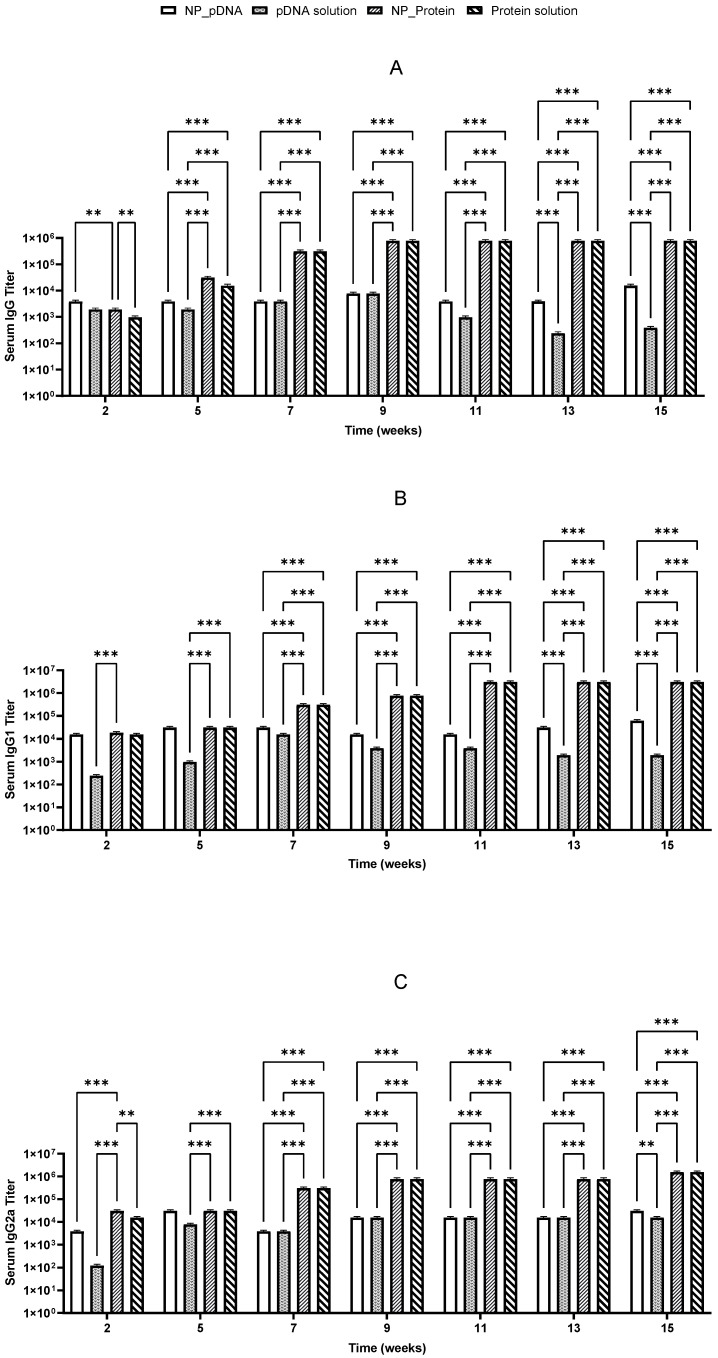
Serum anti-*H. pylori* specific IgG (**A**), IgG1 (**B**), and IgG2a (**C**) titers induced by immunization with pDNA encapsulated (NP_DNA) pDNA in solution and recombinant protein encapsulated (NP_Protein) and recombinant protein in solution by i.m. route. (*n* = 5; mean ± SD) (*** *p* < 0.001 and ** *p* < 0.005).

**Figure 6:**
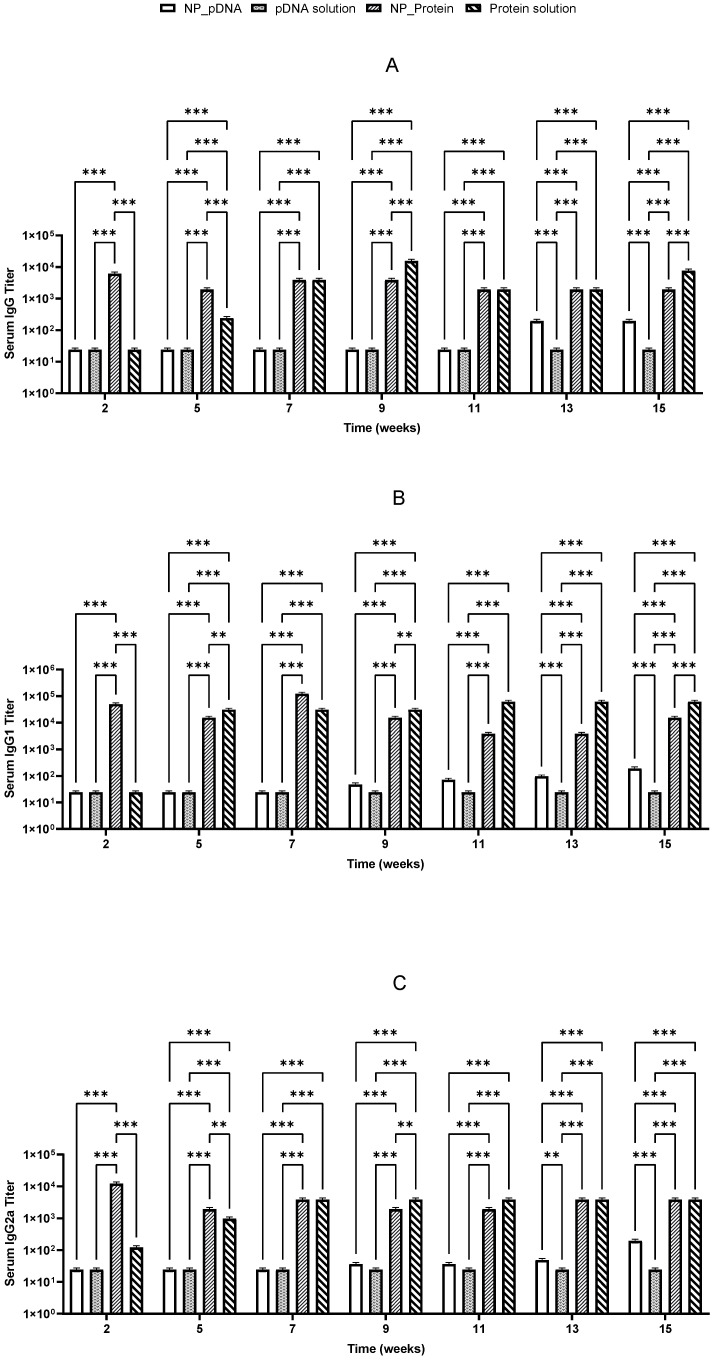
Serum anti-*H. pylori* specific IgG (**A**), IgG1 (**B**), and IgG2a (**C**) titers induced by immunization with pDNA encapsulated into nanoparticles (NP_DNA) pDNA in solution and recombinant protein encapsulated (NP_Protein) and recombinant protein in solution by oral route. (*n* = 5; mean ± SD) (*** *p* < 0.001 and ** *p* < 0.005).

Two weeks after prime vaccination, immunized mice started producing specific IgG antibodies against *H. pylori*. It was also noticed that the booster dose on day 22 induced a significant increase in IgG levels, which remained elevated until the end of the study for almost all vaccinated groups (Figure 5 and Figure 6). High levels of both IgG1 and IgG2a suggest that a balanced immune response was stimulated (Figure 5 and Figure 6).

**Figure 7:**
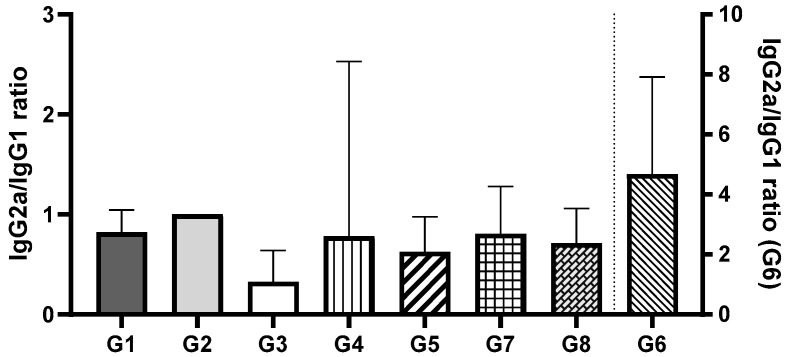
Ratio of serum anti-*H. pylori* specific IgG2a and IgG1 titers after oral and i.m. immunization of BALB/c mice. G1—encapsulated pDNA by oral route; G2—free pDNA in solution by oral route; G3—encapsulated recombinant protein by oral route; G4—free recombinant protein in solution by oral route; G5—encapsulated pDNA by i.m. route; G6—free pDNA in solution by i.m. route; G7—encapsulated recombinant protein by i.m. route; G8—free recombinant protein in solution by i.m. route; (*n* = 5; mean ± SD).

The IgG2a/IgG1 ratio was assessed to evaluate the balance of Th1 and Th2 immune responses (Figure 7). Among all groups, Group 2 demonstrated the most balanced immune response. Comparing the groups that received vaccines via oral administration, no significant differences were observed between Groups 1 and 2 (encapsulated pDNA and pDNA in solution) or between Groups 3 and 4 (encapsulated protein and protein in solution) (*p* > 0.05). However, a statistically significant difference was found between Groups 1 and 2 (pDNA) and Groups 3 and 4 (protein) (*p* < 0.0002). These findings suggest that the orally administered pDNA vaccine, irrespective of the delivery system, induces a more balanced Th1/Th2 immune response compared to the recombinant protein vaccine, which predominantly elicited an IgG1 response, indicative of a Th2 (humoral) response. For groups where the vaccines were administered i.m., no significant differences were observed between Groups 5, 7, and 8 (*p* > 0.05). These groups exhibited a predominance of IgG1, consistent with a Th2 (humoral) immune response. In contrast, Group 6 (pDNA vaccine in solution administered via i.m.) showed a statistically significant difference compared to the other i.m. groups (*p* < 0.005). Group 6 was the only group to exhibit a predominance of IgG2a, indicative of a Th1 (cellular) immune response. Notably, this response aligns with the immune profile typically observed during natural *H. pylori* infection.

Recombinant proteins administered i.m. elicited a robust and balanced immune response, and oral immunization with the same antigens could induce IgG1 predominance, reflecting a Th2-biased response distinct from those induced by natural *H. pylori* infections. Encapsulated recombinant protein did not differ significantly from protein solution, suggesting high immunogenicity of the recombinant protein itself without requiring delivery systems or adjuvants.

Notably, after oral immunization, only the recombinant protein showed an efficient stimulation of specific IgG, IgG1, and IgG2a antibodies, whereas pDNA showed lower IgG isotype titers (Figure 6). Significant differences (*p* < 0.05) were observed between pDNA vaccines and those prepared with *H. pylori* recombinant proteins. These results suggest that the nanoparticulate carriers used may not be suitable for the hostile environment of the stomach, potentially undergoing degradation due to the low pH environment and gastric enzymes.

Both vaccines stimulated Th1 and Th2 immune responses, with the highest responses being triggered by the protein-based vaccines given by the i.m. route. Recombinant protein vaccines induced higher IgG levels compared to pDNA-based vaccines. In general, CS nanoparticles seem to enable a more sustained immune response compared to antigen solutions.

### 3.6. Mucosal IgA Production in Gastric Tissue

**Figure 8:**
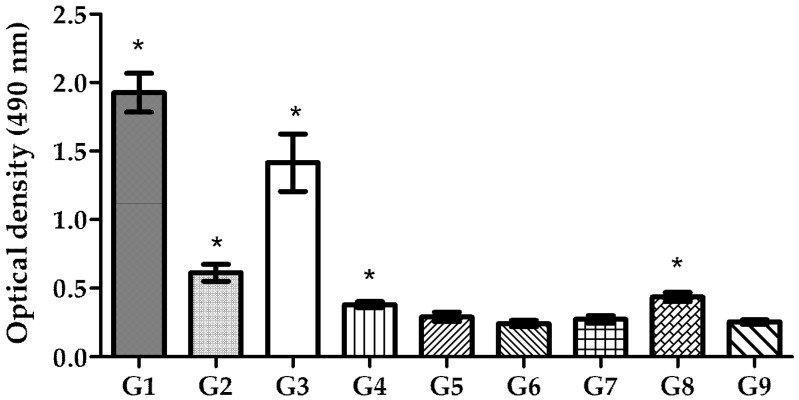
Specific IgA levels in intestine homogenates of mice immunized by i.m. and oral route. G1—encapsulated pDNA by oral route; G2—free pDNA in solution by oral route; G3—encapsulated recombinant protein by oral route; G4—free recombinant protein in solution by oral route; G5—encapsulated pDNA by i.m. route; G6—free pDNA in solution by i.m. route; G7—encapsulated recombinant protein by i.m. route; G8—free recombinant protein in solution by i.m. route; G9—control group (*n* = 5; mean ± SD). (* statistically significant differences (*p* < 0.05) from the control group (G9)).

As *H. pylori* infects the host via the oral route and colonizes the stomach, oral immunization is expected to provide a first line of defense against this bacterium. Studies have shown that the production of local secretory IgA (sIgA) at the mucosal level is critical for establishing this initial defense at the mucosal surface of the stomach, as it promotes lymphocyte proliferation. This process enhances the production of more specific IgA, which blocks *H. pylori* adhesion [36,37]. To assess the success of mucosal immunization, sIgA levels in the intestine of vaccinated BALB/c mice were evaluated (Figure 8). The results demonstrated that mice vaccinated via the oral route exhibited significantly higher levels of local sIgA compared to the control group, with the highest level of sIgA stimulation being obtained in mice that received the pDNA–nanoparticles (*p* < 0.0001). Encapsulated recombinant proteins administered orally also induced a high level of sIgA compared to the negative control (*p* < 0.0001). In contrast, pDNA or recombinant protein solution did not stimulate sIgA production to the same extent as the nanoencapsulated antigens.

### 3.7. Cytokine Production

The immune response stimulated by vaccination can be assessed through cytokines analysis. T-helper (Th) 1 cells mainly secrete IL-2 and IFN-γ, which stimulate a cellular immune response that promotes the synthesis of IgG2a. In contrast, Th2 cells express cytokines such as IL-4, IL-5, IL-6, IL-10, and IL-13, which stimulate a humoral immune response and promote the secretion of IgG1 [38,39].

**Figure 9:**
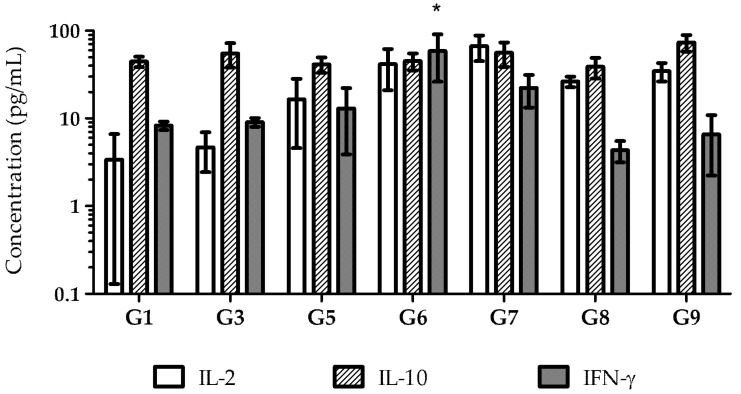
Cytokine levels after splenocyte stimulation with *H. pylori* antigens (Note: G2 and G4 were not analyzed). (*n* = 5; mean ± SD). (* statistically significant differences (*p* < 0.05) from the control group (G9)).

To understand the type of immune response induced by each vaccine formulation and administration route, the supernatants of antigen-stimulated splenocytes from immunized mice were collected, and the cytokines produced in response to each vaccine were quantified (Figure 9). Due to low serum-specific antibody responses, cytokine analysis was not performed on Group 2 (pDNA in solution administered orally) and Group 4 (recombinant proteins in solution administered orally).

All immunized mice developed a humoral immune response, as evidenced by high levels of IL-10, including Group 9, the negative control immunized with empty nanoparticles. These findings suggest that the antigen is immunogenic, capable of inducing both humoral and cellular immune responses, as indicated by the levels of IL-2 and IL-10.

## 4. Discussion

Various vaccination strategies have been developed against *H. pylori*, including the use of whole bacterial lysates [40,41,42], DNA vaccines, and specific recombinant antigens [43]. However, no significant breakthroughs have been achieved. An epitope-based vaccine approach has been proposed as a promising strategy for controlling *H. pylori* infection. Studies have demonstrated that *H. pylori* vaccines comprising a single recombinant antigen had only partial efficacy [44]. Therefore, effective immunity against *H. pylori* is thought to require a combination of antigens involved in the pathogenesis of infection. In this study, fragments of six different *H. pylori* antigens (CagA, UreB, HpaA, VacA, GroEL, and Homb) were selected as candidate antigens to design both a multi-epitope protein-based vaccine and pDNA based-vaccine, aiming to confer protective immunity against various *H. pylori* strains.

To maximize the expression in the *E. coli* system, the method was optimized to determine the optimal induction time and incubation period. According to Olaofe et al. [45], the early exponential phase is the best time to induce protein expression, as cell growth is active, and nutrients are abundant for protein synthesis. However, protein induction often impairs cell growth rates and limits increases in cell mass, which inversely correlates with the synthesis rate of recombinant proteins [46]. Hence, long-time incubation after the induction with IPTG may not be advantageous for increasing expression productivity [47]. Based on these findings, the optimized protocol included the induction with IPTG after 5 h of culture incubation, corresponding to the exponential growth phase, and ending the induction after 3 h of incubation.

In a previous study [16], we demonstrated that CS/Alg nanoparticles protect the encapsulated plasmid DNA from nuclease degradation and achieve high transfection efficacy in various human gastric epithelial cell lines. In line with these findings, plasmid DNA and recombinant protein were encapsulated into CS/DS nanoparticles for i.m. immunization and into CS/Alg/TPP nanoparticles for oral immunization in BALB/c mice. The evaluation of the systemic titers of specific IgG, IgG1, and IgG2a antibodies revealed could suggest a stronger immune response after i.m. immunization. However, the implications of the differences observed between i.m. and oral immunization for protection against *H. pylori*, which primarily affects the GI tract, require further investigation. Local GI immunity, which is more directly influenced by oral immunization, may play a critical role in protection even if serum antibody titers are lower, highlighting the need to consider both local and systemic immunity when evaluating vaccine efficacy.

An effective candidate vaccine against *H. pylori* requires the stimulation of cell-mediated immunity, which plays a crucial role in triggering the secretion of proinflammatory mediators from the innate immune cells, increasing the cytotoxicity mediated by CD8+ T cells, raising the cytokines secretion by CD4+ T cells, and finally inducing macrophage activation and antibody production [48]. Interestingly, *H. pylori* infection primarily elicits a Th1-polarized immune response, leaving the immune system unable to generate a balanced immune response. Therefore, it is thought that an effective therapeutic vaccine should induce a balanced Th1/Th2 immune response to successfully eradicate *H. pylori* infections [37]. Additionally, factors such as the route of administration, dose, and immunological adjuvants significantly influence vaccine effectiveness [49].

In this study, specific IgG isotypes were measured to assess the balance of immune response, characterized by high levels of IgG1 and IgG2a. Both types of vaccines induce immune responses with Th1 and Th2 stimulation, except for the pDNA vaccine administered orally. These results confirm that the delivery route of the pDNA vaccine is important for achieving high immunogenicity of the expressed antigens [50]. Following i.m. vaccination, CS nanoparticles may have contributed to a prolonged presentation of the antigens to antigen-presenting cells, potentially due to their ability to act as a reservoir for the encapsulated pDNA. This prolonged immune response, observed for over 15 weeks, suggests the possibility of a depot effect. However, we acknowledge that other mechanisms, such as vehicle-induced local inflammation or differences in cellular uptake, could also contribute to the sustained immune response. Further studies involving direct measurements of payload release profiles or immunization site depot formation are needed to confirm this hypothesis.

Studies suggest that induction of a well-balanced Th1/Th2 immune response, along with mucosal immunity, could eradicate the infection and confer protection [36,51]. A response of this type was detected by cytokines measurement. In agreement with IgG titer assessment, cytokine levels revealed a more robust immune response after i.m. immunization. Recombinant protein–nanoparticles elicited a well-balanced immune response, confirmed by the presence of the characteristic Th1 and Th2 cytokines. Interestingly, empty CS nanoparticles also induced high levels of IL-2 and IL-10, suggesting the inherent immunostimulatory properties of CS. This observation aligns with previous studies, indicating that CS can act as an adjuvant, enhancing immune responses by stimulating cytokine production and promoting antigen uptake [52]. However, it is important to note that the current study did not include non-specific antigen controls or perform recall antigen assays to assess antigen-specific memory immunity. Therefore, while the observed cytokine profiles indicate an immune response, we cannot conclusively determine the presence of *H. pylori* antigen-specific memory immune cell responses.

Mucosal immunity stimulation includes lymphocyte proliferation, which promotes specific IgA production and blocks *H. pylori* adhesion [37]. sIgA levels tested in the intestines of immunized mice revealed that nanoencapsulated pDNA and recombinant protein, when administered orally, induced high levels of local immunization. In contrast, pDNA and recombinant protein in free solution induced only the secretion of low levels of sIgA. These findings suggest that neither approach can efficiently induce local immunization without a delivery system and adjuvant to simultaneously protect them from degradation and enhance their immunogenicity and uptake by the host cells.

Based on these results, the type of carrier does appear to have a significant influence on the performance of the presented vaccine, particularly in the oral route. The use of CS nanoparticles as a delivery system proved essential for protecting the vaccine and enhancing its efficacy. CS not only serves as a protective carrier but also acts as an immunoadjuvant, contributing to a Th1/Th2 immune response that is balanced or that shifts toward a Th2-dominant response, depending on the load (pDNA or recombinant protein). As previously mentioned, oral vaccines formulated with CS showed higher levels of sIgA. Furthermore, previous studies from our group have demonstrated that the presence of CS enhances cellular transfection efficiency [27], which likely contributes to the observed immune responses. While it is possible that other biocompatible polymers could achieve similar outcomes, this would likely require combining multiple polymers to replicate the unique properties of CS, potentially leading to increased formulation complexity. Thus, CS offers both functional and practical advantages for the development of an effective and efficient vaccine formulation.

## 5. Conclusions

In conclusion, we successfully constructed multi-epitope pDNA and protein-based vaccines against *H. pylori*, which demonstrated the ability to induce an adequate immune response. Oral immunization with vaccines encapsulated in CS nanoparticles stimulated sIgA, suggesting a promising approach for a prophylactic strategy to prevent *H. pylori* colonization in the gastric mucosa. The findings from our mouse model offer valuable insights for advancing vaccine development. However, future studies should evaluate vaccine efficacy, following challenges with diverse *H. pylori* strains, and ideally involve larger animals, such as pigs, to provide more data before clinical studies. While mouse models remain indispensable tools for preclinical assessment, interspecies differences underscore the need for caution when extrapolation results to humans. For example, the mouse and human immune systems share many similarities and often give rise to comparable functional responses, but these responses can be generated by different cell types and pathways. This fact, associated with possible differences in vaccine bioavailability, can overestimate vaccine efficacy, once again highlighting the need for validation across multiple models to ensure translational relevance. Despite these challenges, our work shows promise for a nanoparticle-based vaccine against *H. pylori*.

## Figures and Tables

**Table 1:** Immunization groups with recombinant antigen protein and plasmid by oral and i.m. routes.

Groups	Antigens	Dose/Presentation Antigen	Via
G1 NP_pDNA	Plasmid encapsulated in CS/TPP/Alg nanoparticles	2 × 50 μg/encapsulated in 424 µg of nanoparticles	Oral
G2 pDNA solution	Plasmid in solution	2 × 50 μg/free	
G3 NP_protein	Recombinant protein encapsulated in CS/TPP/Alg nanoparticles	2 × 50 μg/encapsulated in 368 µg of nanoparticles	
G4 Protein solution	Recombinant protein in solution	2 × 50 μg/free	
G5 NP_pDNA	Plasmid encapsulated in CS/DS nanoparticles	2 × 50 μg/encapsulated in 130 µg of nanoparticles	i.m.
G6 pDNA solution	Plasmid in solution	2 × 50 μg/free	
G7 NP_protein	Recombinant protein encapsulated in CS/DS nanoparticles	2 × 50 μg/encapsulated in 75 µg of nanoparticles	
G8 Protein solution	Recombinant protein in solution	2 × 50 μg/free	
G9 NP	CS/DS empty nanoparticles	130 µg of empty nanoparticles	

**Table 2:** Amounts of purified protein obtained before and after buffer exchange and desalting.

Buffer	Protein Concentration (µg/mL)	Total Protein (µg/g) Cells Wet Weight
8 M Urea	310	4.655
100 mM Hepes	129	2.714
Yield	58%	

**Table 3:** Total amount of purified recombinant plasmid (pDNA-CUHpVGHO).

[plasmid]	1769 µg/mL
[plasmid] residual	648 µg/mL
Total [plasmid]	285.5 µg/g cell wet weight

**Table 4:** CS nanoparticles characterization. Values are expressed as mean ± S.D. (*n* = 3).

Nanoparticles		Mean Particle Size (nm)	pDNA/CS (*w*/*w*)	Encapsulation Efficiency (%)
CS/DS	pDNA	242 ± 8	32	100
	Protein	894 ± 6	-	70 ± 10
CS/TPP/Alg	pDNA	195 ± 1	15	100
	protein	328 ± 7	-	70 ± 8

## Data Availability

All data generated or analyzed during this study are included in this published article.
